# First person – Jacob Trend

**DOI:** 10.1242/dmm.052780

**Published:** 2026-01-26

## Abstract

First Person is a series of interviews with the first authors of a selection of papers published in Disease Models & Mechanisms, helping researchers promote themselves alongside their papers. Jacob Trend is first author on ‘ Cortical bone porosity is spatially heterogeneous and VEGF dependent in male bone’, published in DMM. Jacob conducted the research described in this article while a PhD student in Professor Claire Clarkin's lab at University of Southampton, Southampton, UK. He is now a research fellow in the lab of Professor Gavin Foster at University of Southampton, investigating multimodal, multiscale imaging and workflow development.


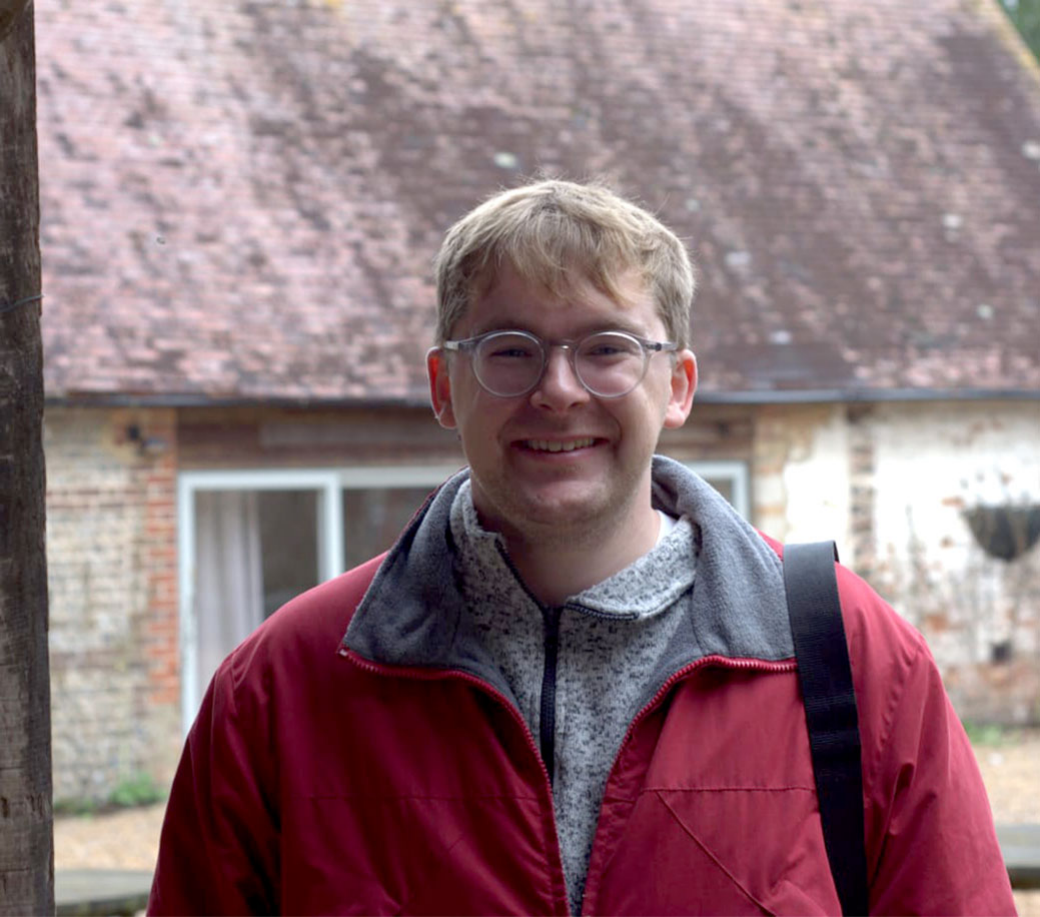


**Jacob Trend**

**Who or what inspired you to become a scientist?**

My A-level teacher – Ms Meigh! She instilled hard work and curiosity – truly lucky to have been taught by such a great teacher.

**What is the main question or challenge in disease biology you are addressing in this paper? How did you go about investigating your question or challenge?**

In this work, I address the question of how the spatial organisation of cortical porosity – specifically intracortical vascular canals and osteocyte lacunae – contributes to regional vulnerability of bone to structural deterioration and fracture. Although increased cortical porosity is a key feature of degenerative bone diseases, we lack a detailed understanding of how vascular and osteocyte compartments are organised in 3D within different regions of the cortex and how this organisation is maintained or disrupted in disease. To investigate this, I focused on the murine tibiofibular junction, a site composed entirely of cortical bone and previously shown to exhibit region-specific changes in vascular canals. I used synchrotron X-ray computed tomography to image the cortical microarchitecture at high resolution, and subsequently developed an automated regionalisation workflow to segment the cortex into anterior, posterior, lateral and medial quadrants, minimising observer bias. From this, we quantified cortical porosity, intracortical canal morphology, and osteocyte lacunar number and volume within each region. We also developed and implemented a 3D lacunar distance mapping approach, enabling measurement of the minimum distance of each osteocyte lacuna to potential vascular supply (bone surfaces and intracortical canals) and classification of lacunae as canal associated or surface associated. Finally, we applied this framework to osteocalcin-specific VEGF knockout mice, a model with altered bone vascularisation and mineralisation, to determine how loss of osteoblast-derived VEGF affects vascular–lacunar spatial organisation. Together, these methods allowed us to define region-specific vascular–osteocyte architectures in health and assess how they are perturbed in a pathological context.

[Our] methods allowed us to define region-specific vascular–osteocyte architectures in health and assess how they are perturbed in a pathological context

**How would you explain the main findings of your paper to non-scientific family and friends?**

Bones might look solid from the outside, but inside the hard outer shell there is a very fine network of micron-scale channels and pores. The channels house blood vessels and nerves, and the small pores contain a network of bone cells that help maintain and repair the bone. In my study, I found that this internal network is not the same everywhere in the bone. In one particular unique region, there are more of these channels, and the nearby bone cells sit in larger pores. I then looked at mice in which a key signal that helps blood vessels and bone cells communicate in bone (called VEGF) was removed from certain bone-forming cells. In these mice, the normal pattern – where this unique region has a special arrangement of blood channels and larger cell spaces – was lost. The bone became more porous overall, and the clear relationship between the blood channels and the bone cells was disrupted.

**What are the potential implications of these results for disease biology and the possible impact on patients?**

Our findings indicate that the posterior cortex at the tibiofibular junction represents a region with uniquely high cortical porosity, elevated canal density and enlarged canal-associated osteocyte lacunae, suggesting a specialised microenvironment that is particularly dependent on intracortical vasculature for osteocyte support. This has several implications for disease biology.

First, the data support the concept that specific cortical regions may be intrinsically more susceptible to pathological disruption, especially in conditions where vascular supply or osteocyte function is compromised. Such regional vulnerability may contribute to the uneven distribution of cortical fragility and fracture risk observed in disorders such as osteoporosis.

Second, the work identifies osteoblast-derived VEGF as a key regulator of spatial coupling between intracortical vasculature and osteocytes. Loss of VEGF signalling leads to homogenisation of cortical porosity, loss of region-specific canal–lacunae organisation and mineralisation deficits. These findings suggest that impaired VEGF pathways could play a mechanistic role in the microarchitectural deterioration seen in human skeletal disease.

Finally, the results raise the possibility that therapeutic strategies may need to target microregional bone environments, rather than focus solely on increasing total bone mass. Preserving or restoring the local osteocyte–vascular architecture – particularly in regions with high metabolic or structural demand – may be crucial for maintaining cortical integrity.

For patients, a deeper understanding of these spatial relationships may contribute to more precise diagnostic markers of bone quality, extending beyond conventional bone density measures, and may guide the development of more targeted interventions aimed at stabilising the vascular–osteocyte network in regions most critical for preventing structural failure and fracture.

**Figure:**
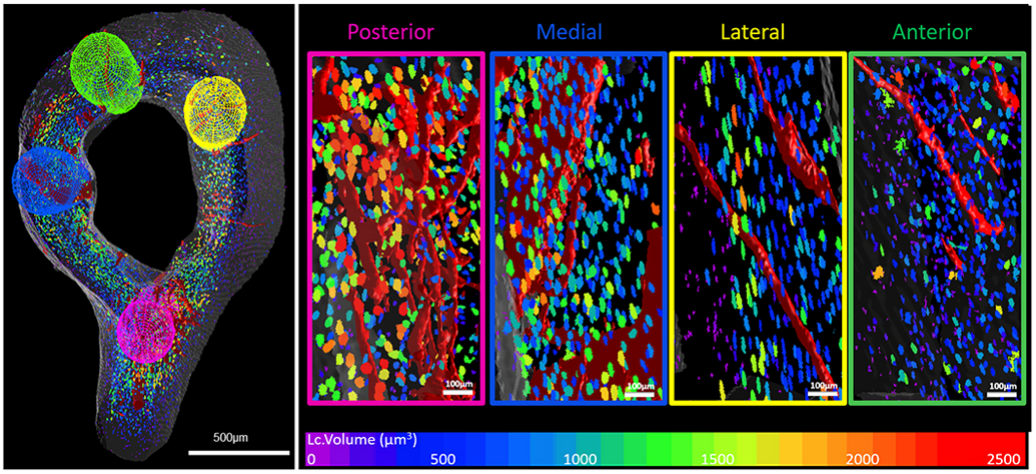
**Vascular–lacunar volume mapping: a tool for the study of allied microstructural networks.** Regional assessment of murine bone reveals heterogeneity between bone reigns – here, we showcase how osteocyte lacunar (Lc.) volume varies as a function of bone region and proximity to intracortical canals.

**Why did you choose DMM for your paper?**

DMM is an appropriate venue for this study because my work directly links advanced imaging of bone microarchitecture with a genetically defined disease model to uncover mechanisms underlying cortical bone fragility. Although the paper begins by characterising healthy bone, the central aim is mechanistic: to understand how regional interactions between osteocytes and the intracortical vasculature are established, maintained and disrupted in disease. By incorporating the osteocalcin-specific VEGF knockout – an established model of impaired bone vascularisation and mineralisation – I show how loss of a specific molecular pathway alters cortical porosity, osteocyte–vascular coupling and spatial heterogeneity. This provides mechanistic insight into processes that mirror features of human bone fragility disorders such as osteoporosis.

**Given your current role, what challenges do you face and what changes could improve the professional lives of other scientists in this role?**

Current challenges for me centre on the development and adaption of new imaging workflows, often integrating methodologies from differing spheres of research. I hope that an improved publication of multi-modal workflows, and sharing of plugins, macros and pipelines in open access environments, will help overcome some of these challenges.

**What's next for you?**

I am completing a postdoctoral research position in the 4D, multimodel assessment of coral biomineralisation, with a focus on transferring techniques from bone research to the coral community.

**Tell us something interesting about yourself that wouldn't be on your CV**

One thing that wouldn't appear on my CV is how much of my scientific motivation comes from the methods themselves. I am genuinely driven by imaging and analysis – especially developing workflows that allow us to visualise or quantify structures in ways we couldn't before.
